# Acute cytotoxic effects of silica microparticles used for coating of plastic blood-collection tubes on human periosteal cells

**DOI:** 10.1007/s10266-020-00486-z

**Published:** 2020-01-30

**Authors:** Hideo Masuki, Kazushige Isobe, Hideo Kawabata, Tetsuhiro Tsujino, Sadahiro Yamaguchi, Taisuke Watanabe, Atsushi Sato, Hachidai Aizawa, Carlos Fernando Mourão, Tomoyuki Kawase

**Affiliations:** 1 Tokyo Plastic Dental Society, 2-262-2 Oji, Kita-ku, Tokyo, 114-0002 Japan; 2 Department of Oral Surgery, Dentistry School, Fluminense Federal University, Rio de Janeiro, Brazil; 3 Division of Oral Bioengineering, Institute of Medical and Dental Sciences, Niigata University, 2-5274 Gakkocho-dori, Chuo-ku, Niigata 951-8514 Japan

**Keywords:** Platelet-rich fibrin, Silica, Cytotoxicity, Apoptosis, Periosteal cells

## Abstract

Because of its simple operation, platelet-rich fibrin (PRF) is becoming more popular than the original form, platelet-rich plasma (PRP), in regenerative dentistry. PRF preparation requires plain glass blood-collection tubes, but not either anticoagulants or coagulation factors. However, such glass tubes designed for laboratory testing are no longer commercially available. Although several glass tubes specifically designed for PRF preparation are available, many clinicians prefer to obtain stably supplied substitutes, such as silica-coated plastic tubes produced by major medical device companies. The quality of PRF prepared by silica-coated tubes has not been assessed and we previously reported significant contamination of silica microparticles in the resulting PRF matrix and alerted clinicians against the use for PRF preparation. To further assess the biosafety of the silica microparticles, we presently examined their effects on human normal periosteal cells derived from alveolar bone. The periosteal cells were obtained from explant cultures of small periosteal tissues obtained from healthy donors. Silica microparticles were obtained from silica-coated tubes and added to cell cultures. Cellular responses were monitored using a tetrazolium assay, phase-contract inverted microscopy, an immunofluorescence method, and scanning electron microscopy. Silica microparticles adsorbed onto the cell surface with seemingly high affinity and induced apoptosis, resulting in significant reduction of cell proliferation and viability. These findings suggest that silica microparticles contained in plastic tubes for the purpose of blood coagulation are hazardous for various cell types around sites where silica-contaminated PRF matrices are implanted.

**Electronic supplementary material:**

The online version of this article (10.1007/s10266-020-00486-z) contains supplementary material, which is available to authorized users.

## Introduction

Due to their high cost–performance ratio, platelet concentrates, such as platelet-rich plasma (PRP), have been increasingly and widely applied in regenerative medicine. Among the types of PRP and their derivatives, platelet-rich fibrin (PRF) has been increasingly used, especially in the field of regenerative dentistry, in the past several years, since PRF preparation is user-friendly and less costly. PRF preparation requires only a plain glass tube to activate the intrinsic coagulation pathway to form a fibrin clot [1]. However, this simple prerequisite has ironically proven problematic in the clinical setting, since the production of plain glass tubes has been discontinued by major medical device manufacturers, restricting a stable supply of the tubes for clinicians [2, 3]. Instead, based on information from local distributors and/or other clinicians, they tend to use silica-coated plastic blood collection tubes, as the tubes are produced by major manufacturers and are readily available.

The necessity for glass tubes and the alternative use of silica-coated tubes are explained by the activation of coagulation factor XII by the negatively charged silanol groups on the glass surface [4]. The surface of silica, which is a major component of glass, is also negatively charged. Thus, it can be substituted for glass. As an additional benefit, silica microparticles used for surface coating can be easily detached upon blood collection and act ubiquitously to activate the coagulation cascade more efficiently than glass [3]. Thus, silica-coated tubes have become routinely used for serum testing.

Silica dusts, especially those composed of crystalline silica particles, cause lung silicosis and lung cancer [5]. Compared with crystalline silica, amorphous silica has generally been thought to be less hazardous [6] and has become widely used in various industrial products, such as additives in varnishes, paints, and glues. In addition, amorphous silica preparations are used in the production of free-flowing powders for food stuffs, animal feeds, pharmaceuticals, and cosmetics, etc. However, recently, increasing numbers of studies have demonstrated that amorphous silica induces toxic effects on cultured cells [7–9]. The mechanism of its cytotoxicity is thought to be injury of the plasma membrane by silica-dependent production of reactive oxygen species [8, 10].

To our knowledge, only one manufacturer has disclosed that the silica particles are amorphous. Considering its reduced toxicity, we think that amorphous silica microparticles are used for the coating in this type of tube. However, such silica-coated tubes were originally designed for laboratory testing and were approved as products for laboratory use only by individual countries’ regulatory authorities. The use of the tubes for PRF therapy can be questioned. To address this concern, in a previous study [2], we demonstrated using spectrophotometric and microscopic methods that silica microparticles detached from the inner wall are immediately incorporated into the PRF matrix.

Judging from the accumulated data [7–9], our previous data are sufficient to indicate that those silica microparticles are topically hazardous in our bodies. To provide absolutely convincing evidence, we performed the present study that examined the possible health hazard caused by silica microparticles derived from PRF matrix.

## Materials and methods

### Culture of human periosteal cells

Two patients (24-year-old male and 22-year-old female) requiring wisdom tooth extraction participated in this study after providing written informed consent. Aliquots of periosteum tissues were aseptically dissected from the buccal side of the retromolar region in the mandible of healthy donors, cut into small segments, and cultured in MSC-PCM medium (Kohjin Bio, Sakado, Japan) supplemented with 4% fetal bovine serum (FBS; Thermo Fisher Scientific, Waltham, MA, USA) for 4 weeks [11]. The resulting periosteal sheets were digested with 0.05% trypsin and 0.53 mM EDTA. The dispersed single cells were further expanded in MSC-PCM medium (Kohjin Bio, Sakado, Japan) supplemented with 10% FBS. The cells were frozen in liquid nitrogen until use.

The study design and consent forms for all the procedures (project identification code: 2015-2143) were approved by the Ethics Committee for Human Subjects of the Niigata University School of Medicine (Niigata, Japan) on 12 June, 2017, in accordance with the Helsinki Declaration of 1964 as revised in 2013.

### Preparation of suspension of silica microparticles

Each silica-containing tube was filled with 7 mL of MSC-PCM containing 10% FBS and vortexed to fully detach the silica microparticles from the inner wall or film. The resulting silica suspensions were stored at 4 ℃ until use (< 1 week). Before use, silica suspensions were well vortexed and serially diluted with the same FBS-containing medium before use. Regarding individual differences in silica contents in the same products of the same lots, as far as we examined using a spectrophotometer, the differences ranged within 10%.

Based on the previous findings [2], the sizes of silica microparticles in individual tubes are roughly determined. Neotube: several microns to 40 μm, Vacuette: submicrons to 20 μm, Venoject II: submicrons to 6 μm.

As the negative control, Cytrans Granules (14 mg) were crushed by a Coolmill freeze crusher (Tokken, Inc., Kashiwa, Japan) and suspended in the medium. Cytrans Granules, which are bone graft substitutes composed of carbonate apatite, have been demonstrated to be of high biocompatibility in preclinical studies and subsequent clinical trials, and were recently approved by Japan’s regulatory agency (Pharmaceuticals and Medical Devices Agency) in the category of Class VI medical device/material [12–14]. They are awaiting US Food and Drug Administration (FDA) approval. Therefore, we thought that this biologically safe biomaterial is good enough to highlight the cytotoxicity of silica microparticles contained in silica-coated tubes that are usually approved as a Class II (or I) medical device by major countries’ or regions’ regulatory agencies.

### Cell growth/viability assay using cell counting kit-8

Periosteal cells were seeded at a density of 1 × 10^4^ cells/well in a 24-well plate and treated for 3 days with silica microparticles diluted in MSC-PCM containing 10% FBS. At the end of culture, the medium was replaced with Hank’s balanced salt solution (HBSS) containing 10% formazan solution of the cell counting kit-8 (Dojin, Kumamoto, Japan). The cells were further incubated for 1 h in a CO_2_ incubator. The HBSS was transferred into 96-well plates and absorbance was measured at 450 nm using a model 680 plate reader (Bio-Rad, Hercules, CA, USA).

In parallel, phase-contrast images of the cells were also photographed using an Eclipse Ti-U inverted microscope (Nikon, Tokyo, Japan) at 24, 48, and 72 h of culture.

### Scanning electron microscopy (SEM) examination

Periosteal cells were seeded at a density of 8 × 10^4^ cells in a 35 mm-diameter dish and incubated with silica microparticles diluted 1:8 for 1–3 days, fixed with 2.5% neutralized glutaraldehyde, dehydrated, and freeze-dried as described previously [2, 15]. The rim of the dish was removed and examined by SEM) using a TM-1000 microscope (Hitachi, Tokyo, Japan) at an accelerating voltage of 15 kV.

### Time-lapse recording of cell migration

For time-lapse recording, the stage incubation chamber connected to a CO_2_, humidity and temperature controller (5% CO_2_, UNO; Okolab S.r.l., Naples, Italy) was set up on the stage of an inverted microscope [16]. The 35 mm-diameter dish in which the periosteal cells and silica microparticles were placed with culture medium was set into the chamber and the time-lapse recording was started. The time-lapse recording system consisted of a QIClick monochrome CCD camera (Nippon Roper, Tokyo, Japan) and an Endeavour AT992E personal desktop computer (EPSON, Suwa, Japan) equipped with VisiView imaging software (Visitron Systems GmbH, Puchheim Germany). The phase-contrast images were obtained at 10-min intervals for 24 h. The data were saved as an AVI file and then converted to an MP4 file.

### Detection of apoptosis

Periosteal cells prepared as described above were fixed with 10% formalin and treated with phycoerythrin (PE)-conjugated Annexin V (BioLegend, San Diego, CA, USA) and fluorescein isothiocyanate (FITC)-conjugated phalloidin (Abcam, Cambridge, MA, USA) according to the manufacturers’ instructions. It is noted that Annexin V can detect phosphatidylserine in live or fixed cells [17]. The cells were examined am Eclipse 80i fluorescence microscope (Nikon).

### Statistical analyses

The data are expressed as mean ± standard deviation. For multigroup comparisons, statistical analyses were performed to compare the mean values by the Kruskal–Wallis one-way analysis of variance, followed by a Steel–Dwass multiple comparisons test (BellCurve for Excel; Social Survey Research Information Co., Ltd., Tokyo, Japan). Differences with *P* values < 0.05 were considered significant.

## Results

**Fig. 1:**
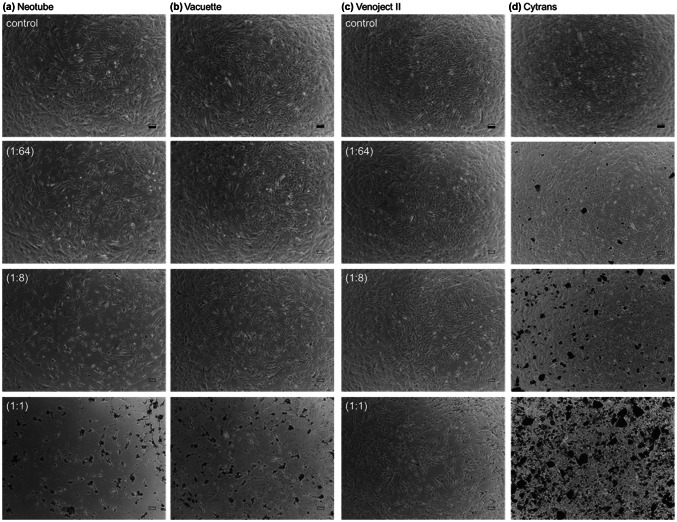
Phase-contrast images of human periosteal cells treated with silica microparticles. The cells were treated with silica microparticles derived from **a** Neotube, **b** Vacuette, or **c** Venoject II for 72 h. Cells were photographed without fixation. As the negative control, cells were treated with **d** synthetic carbonate apatite particles (Cytrans Granules). Values in parentheses represent dilution. Scale bar is 100 µm

The effects of silica microparticles on the cell appearance and the apparent cell density were initially examined. Phase-contrast images of human periosteal cells treated with silica microparticles are shown in Fig. 1. As the dilution of silica suspension was reduced, the cell density became increasingly sparse and the spindle-like shape of cells disappeared similarly in (a) Neotube, (b) Vacuette and (c) Venoject II. In contrast, (d) Cytrans Granules did not substantially reduce cell density or change cell appearance. These data were confirmed by the additional experiment using different donor-derived periosteal cells.

**Fig. 2:**
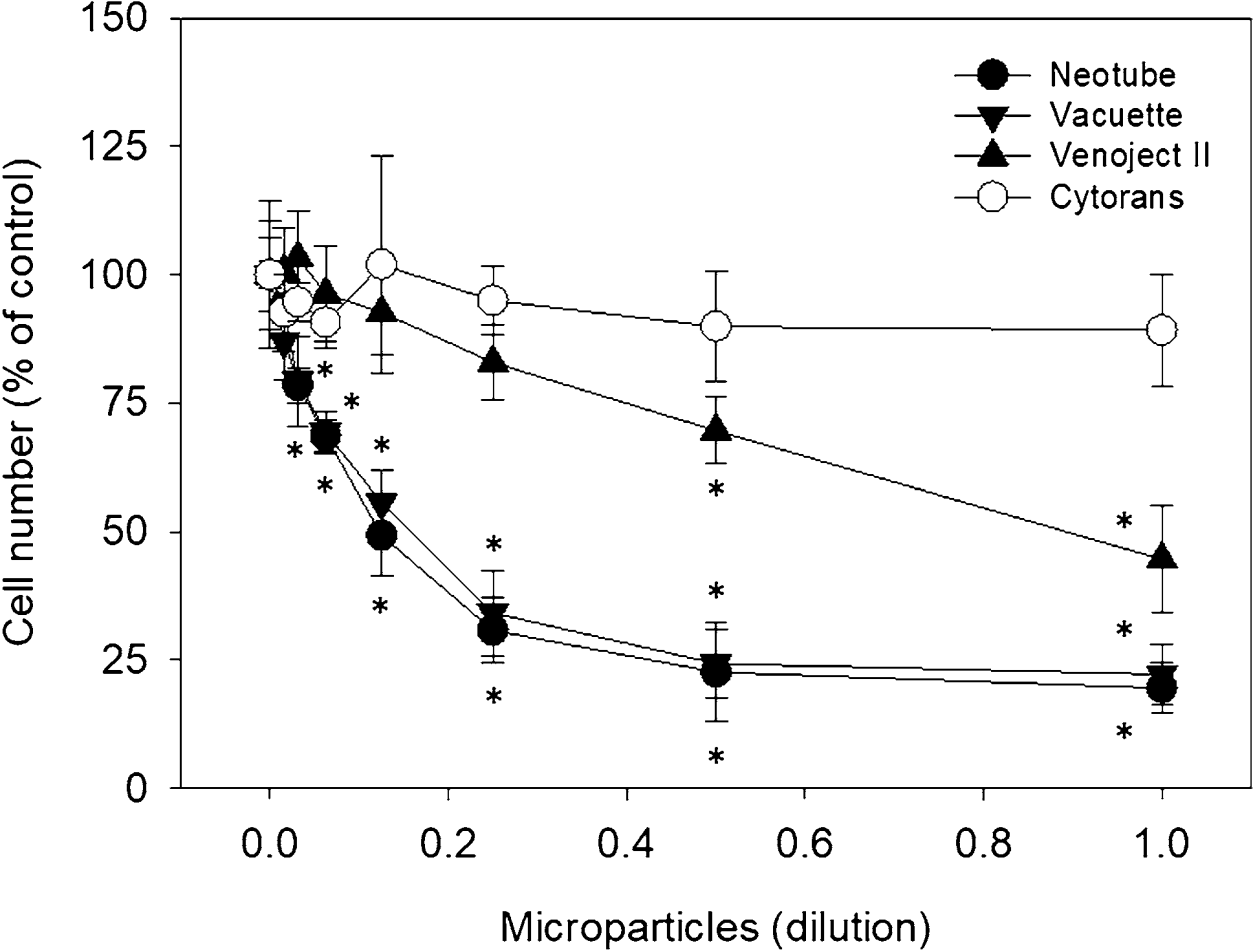
Effects of silica microparticles on the proliferation and viability of human periosteal cells. The cells were seeded into wells of 24-well plates and treated with silica microparticles for 72 h. Cell numbers were assessed using a cell counting kit-8, and the absorbance was measured at 450 nm. Data were obtained from six samples (*N* = 6) of two representative experiments using periosteal cells derived from two independent donors. Asterisks represent *P* < 0.05 compared with the negative control, Cytrans Granules

These semi-quantitative findings were quantitated using the formazan assay. Effects of silica microparticles on the proliferation and viability of human periosteal cells are shown in Fig. 2. In comparison with the negative control, Cytrans Granules, silica microparticles derived from Neotube and Vacuette significantly reduced cell viability at the dilution of 1:32 and 1:16, respectively. Since Venoject II contains fewer silica microparticles per tube [2], its silica particles exerted growth inhibitory effects at lower dilutions (1:4 and lower). However, when not diluted, all the silica microparticles substantially inhibited growth.

**Fig. 3:**
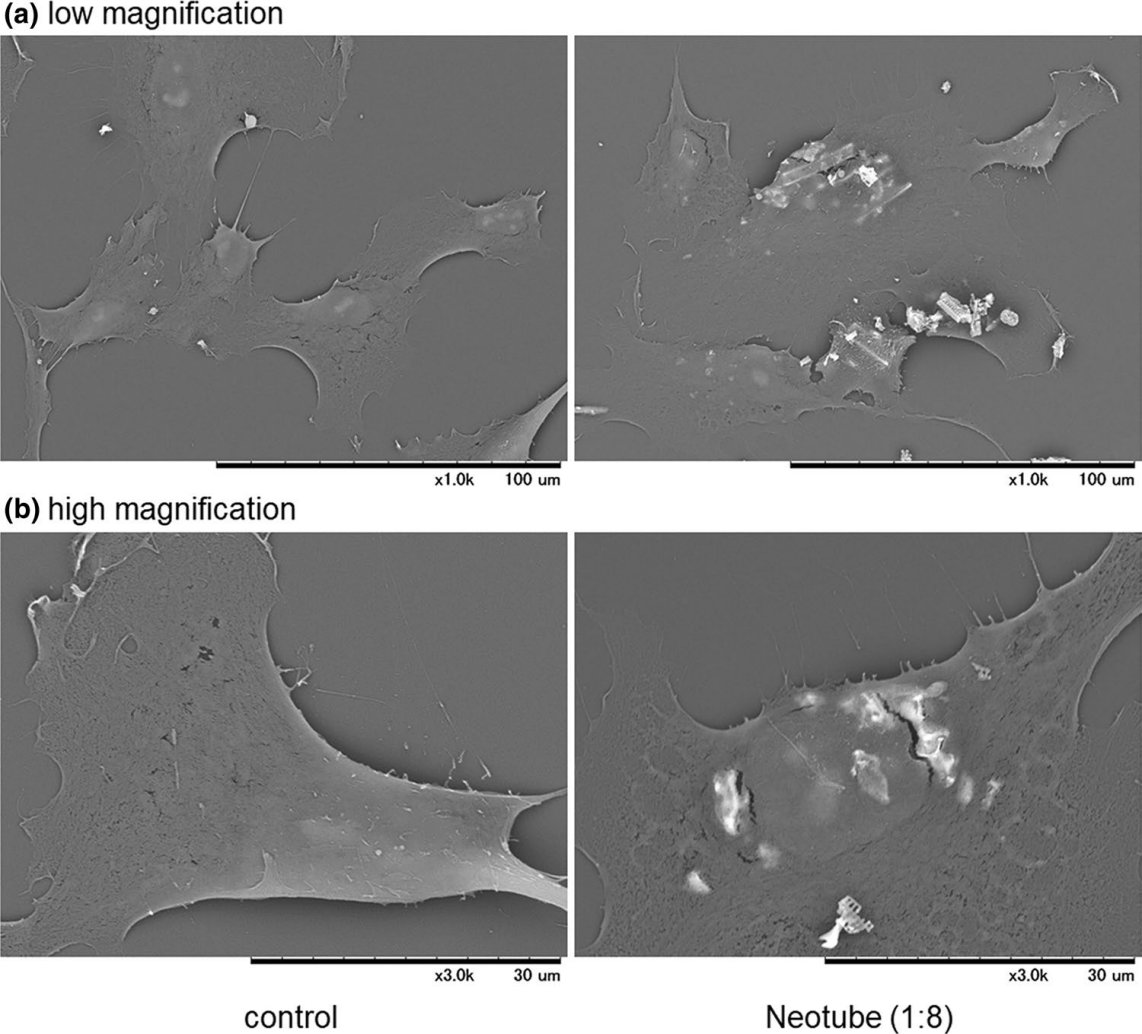
Microstructural images of human periosteal cells treated with silica microparticles. The cells were treated with silica microparticles derived from Neotube (1:8 dilution) for 24 h, fixed, and examined using SEM at **a** low magnification and **b** high magnification. Similar observations were obtained from four other independent experiments, including Vacuette’s silica, using periosteal cells derived from different donors

Microstructural images of human periosteal cells treated with silica microparticles are shown in Fig. 3. Silica microparticles derived from Neotube seemed to be adsorbed nonspecifically on the plasma membrane of periosteal cells and some seemed to be incorporated into the cytoplasm.

**Fig. 4:**
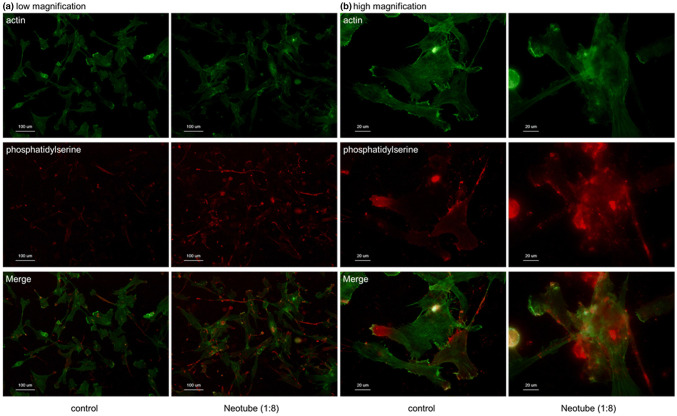
Fluorescence visualization of apoptosis in human periosteal cells treated with silica microparticles. The cells were treated with silica microparticles derived from Neotube for 24 h. The fixed cells were probed with PE-conjugated Annexin V for detection of phosphatidylserine on cell surface, which is accepted as a marker of apoptosis at **a** low magnification and **b** high magnification. The cells were counterstained with FITC-conjugated phalloidin to visualize cytoskeletal polymerized actin. Similar observations were obtained from four other independent experiments, including Vacuette’s silica, using periosteal cells derived from different donors

Since cytotoxic effects of silica microparticles are thought to be mediated by reactive oxygen species [8], the findings indicating the contact of silica microparticles and the plasma membrane were suggestive of membrane disruption and subsequent cell death. Fluorescence visualization of apoptosis in human periosteal cells treated with silica microparticles is shown in Fig. 4. Silica microparticles were coincidently visualized by PR-conjugated Annexin V; however, apoptotic cells were generally stained mildly. Treatment with silica microparticles increased the number of Annexin V-stained cells.

**Fig. 5:**
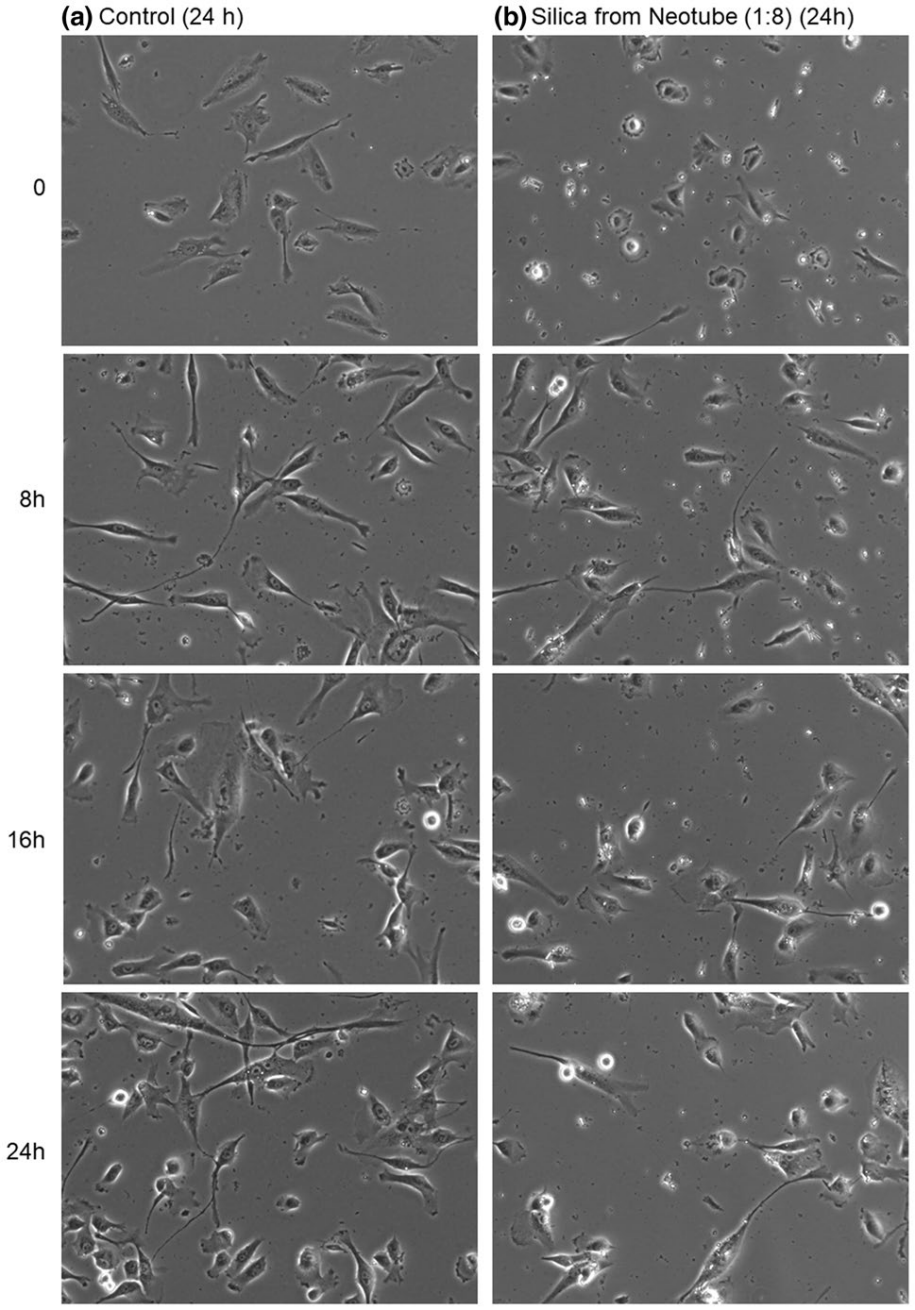
Effects of silica microparticles on the migration and density of human periosteal cells. The cells were treated without silica microparticles derived from Neotube and subjected to time-lapse recording for 24 h. Photomicrographs acquired at 0, 8, 16, and 24 h are shown. Similar observations were obtained from four other independent experiments, including Vacuette’s silica, using periosteal cells derived from different donors

To obtain additional data supporting these findings, we performed time-lapse recording of cell behavior. Effects of silica microparticles on the migration of human periosteal cells are shown in Video 1. In the control culture without silica microparticles, periosteal cells migrated actively and divided. In contrast, cells treated with silica microparticles derived from Neotube migrated like carriers of silica microparticles and less actively than the control cells. Cell division seemed to be suppressed. These video data are summarized by capturing photomicrographs at 0, 8, 16, and 24 h (Fig. 5).

## Discussion

In this study, we obtained clear evidence that silica microparticles derived from commercially available blood collection tubes exert toxic effects on human periosteal cells by adsorbing on the plasma membrane and inducing apoptosis. In addition, this cytotoxicity exceeded our prediction and silica microparticles contained in silica-coated tubes (e.g., Neotube and Vacuette) were sufficient to completely disrupt cell growth and viability in a dose (dilution)-dependent manner. We performed the cytotoxicity experiments using human periosteal cells derived only from two donors. However, these cells were normal cells similar to primary cultures and their chromosome abnormality was not detected in the routine laboratory testing for clinical use. Taken together with the widely accepted evidence of the toxicity of silica particles [8], regardless of the sample size, our data can be considered sufficient to exclude the myth that silica-coated plastic tubes can be used for PRF preparation as a safe substitute for conventional glass tubes. Since legal or biomedical limits on silica particles contaminated in PRF matrices are not established by individual countries’ regulatory agencies or the World Health Organization, PRF users should pay special attention to the present findings and recognize the possibility that PRF matrices prepared by using such silica-coated tubes are hazardous to patients’ health.

Amorphous silica is less toxic than crystalline silica and so has been used for many industrial products. However, it has increasingly been reported that amorphous silica is also hazardous to our health. In our previous study [2], we demonstrated that 5–30% silica microparticles, depending on tube brands, can be included into the resulting PRF matrix. The collective data support the prediction that PRF preparations using silica-coated tubes could be toxic to the surrounding cells at implantation sites. During and after preparation of PRF matrix, silica microparticles may also over-activate or disrupt platelets [18] and other blood cells in the PRF matrix to reduce its therapeutic potency and efficacy.

To our knowledge, there have been no reports of severe complications from the application of silica-dependent PRF preparations. This is probably due to efficient clearance of those microparticles by phagocytosis or extrusion, detoxication by scavengers [19], and/or cytoprotection by redox systems [20] and serum albumin [21]. Thus, even if complications arise, they may be only marginally, if at all, delay tissue regeneration or may only slightly exacerbate inflammation. Lung silicosis is caused by chronic inhalation of silica dusts for a prolonged period of time [22], whereas PRF matrices are usually implanted once in soft tissue regenerative therapy and bone augmentation prior to dental implant therapy. In addition, the history of the clinical use of such PRF matrices is much shorter (only the past several years) than that of lung silicosis (several decades). Thus, accumulation of DNA damage in cells as observed in silicosis-derived lung cancer [22] likely does not occur in cells involved in regenerative dentistry. As discussed above, severe complications may not be likely. However, since the use of silica-coated tubes has no biomedical merits, we recommend clinicians not use this type of blood-collection tube for PRF preparation.

To avoid misunderstanding, it must be noted that silica is different from silicone. In fact, a historical debate may have arisen because of this misunderstanding [23, 24]. Furthermore, when our previous study was published [2], we received some confusing comments. The web site provided by Steam Peak International concisely summarizes the terminology regarding silica, silicon, and silicone [25]. According to this web site, silica, which is also known as silicon dioxide, is a compound that naturally forms in the reaction between oxygen and silicon. Silica is commonly used in the manufacturing of glass, ceramics, optical fiber, and cement. Silicon (Si) is the second most abundant element on Earth. However, it is rarely found in its original state as Si as it readily reacts with oxygen to form mainly silicon dioxide. In contrast, silicone is a synthetic polymer created from the combination of silicon, oxygen, carbon, and/or hydrogen. Unlike natural materials that include silica and silicon, silicone is a man-made product that is manufactured in factories as a solid, liquid, and gel. Silicone is commonly used as a sealant, electrical insulation, component of cooking utensils, and as a coating of test tubes.

Therefore, even though silicone used for tube coating may contain silica-like compounds, it cannot activate blood coagulation. Excess silicone-coating actually delays coagulation. Furthermore, even if silicone has negative effects on the immune system and/or cells directly involved in tissue regeneration, these effects should be distinguished from those of silica. In any case, when platelet concentrates are prepared for use of regenerative therapy, we believe that real “plain” tubes that are approved by regulatory authorities of individual countries, regardless of their original materials, are better to use.

## Conclusions

Commercially available silica-coated blood-collection tubes contain cytotoxic silica microparticles. These silica microparticles are incorporated into the resulting PRF matrix and implanted for regeneration and repair of injured tissues. Thus, it is plausible to suggest that tissue regeneration could be hampered or disrupted. Even though severe complications have not been reported, we still provide this data to alert clinicians not to use this type of bold-collection tubes for PRF preparation.

## Electronic supplementary material

Below is the link to the electronic supplementary material.

Video 1 Effects of silica microparticles on the migration of human periosteal cells. The cells were treated without silica microparticles derived from Neotube and subjected to time-lapse recording for 24 h (control). (MP4 3650 kb)

Video 2 Effects of silica microparticles on the migration of human periosteal cells. The cells were treated with silica microparticles derived from Neotube and subjected to time-lapse recording for 24 h (silica-treated). (MP4 3449 kb)
